# *TREX1* 531C/T Polymorphism and Autoantibodies Associated with the Immune Status of HIV-1-Infected Individuals

**DOI:** 10.3390/ijms24119660

**Published:** 2023-06-02

**Authors:** Maria Alice Freitas Queiroz, Tuane Carolina Ferreira Moura, Carlos David Araújo Bichara, Lorena Leticia Peixoto de Lima, Allysson Quintino Tenório de Oliveira, Ranilda Gama de Souza, Samara Tatielle Monteiro Gomes, Ednelza da Silva Graça Amoras, Antonio Carlos Rosário Vallinoto

**Affiliations:** 1 Laboratory of Virology, Institute of Biological Sciences, Federal University of Pará, Belém 66075-110, Brazil; tuanecfmoura@gmail.com (T.C.F.M.); bichara@amaralcosta.com.br (C.D.A.B.); lorenalplima@gmail.com (L.L.P.d.L.); allyssonquintino@yahoo.com.br (A.Q.T.d.O.); ranilda@ufpa.br (R.G.d.S.); samara_tatielle@yahoo.com.br (S.T.M.G.); ednelza@hotmail.comr (E.d.S.G.A.); vallinoto@ufpa.br (A.C.R.V.); 2 Graduate Program in Biology of Infectious and Parasitic Agents, Institute of Biological Sciences, Federal University of Pará, Belém 66075-110, Brazil

**Keywords:** HIV-1, TREX-1, polymorphism, ART, autoantibodies

## Abstract

Autoimmune diseases can develop during HIV-1 infection, mainly related to the individual’s immune competence. The study investigated the association of the *TREX1* 531C/T polymorphism and antinuclear antibodies (ANA) in HIV-1 infection and the time of antiretroviral therapy (ART) used. Cross-sectional and longitudinal assessments were carried out in 150 individuals, divided into three groups: ART-naïve, 5 years and 10 years on ART; ART-naïve individuals were evaluated for 2 years after initiation of treatment. The individuals’ blood samples were submitted to indirect immunofluorescence tests, real-time PCR and flow cytometry. The *TREX1* 531C/T polymorphism was associated with higher levels of TCD4^+^ lymphocytes and IFN-α in individuals with HIV-1. Individuals on ART had a higher frequency of ANA, higher levels of T CD4^+^ lymphocytes, a higher ratio of T CD4^+^/CD8^+^ lymphocytes and higher levels of IFN-α than therapy-naïve individuals (*p* < 0.05). The *TREX1* 531C/T polymorphism was associated with better maintenance of the immune status of individuals with HIV-1 and ANA with immune restoration in individuals on ART, indicating the need to identify individuals at risk of developing an autoimmune disease.

## 1. Introduction

Human immunodeficiency virus and acquired immunodeficiency syndrome (HIV/AIDS) remain serious health problems worldwide, responsible for the deaths of more than 40 million people, with estimates of approximately 38 million people living with the infection [1]. The introduction of antiretroviral therapy (ART) promoted a significant reduction in the morbidity and mortality rate caused by HIV-1 and has contributed to changing the scenario of the infection, which is now considered a chronic condition [2]. However, although ART has had a significant impact on infection control, its use needs to be monitored to assess possible intercurrences, including metabolic changes [3,4]. Prolonged use of ART has been associated with the development of autoimmune conditions, as ART, by promoting an improvement in the immune status, can induce different autoimmune reactions [5,6,7].

The development of autoimmune diseases still has no defined cause, but genetic, environmental and regulatory factors of the immune system have been strongly associated with the diseases [8]. Among the environmental factors that can induce autoimmunity are infectious diseases [9]. In HIV-1 infection, autoimmune diseases can develop in different stages; however, they are more evident in the period in which there is maintenance and restoration of immune competence resulting from the use of ART [5]. The main processes that can induce autoimmune diseases in these individuals include molecular mimicry, dysregulation of the interaction of B and T lymphocytes and the activation of polyclonal B lymphocytes, which can favor the synthesis of autoantibodies [5,10].

Some components of immunity act to maintain the individual’s immune tolerance, degrading cytosolic DNA; TREX-1 is the main DNA exonuclease in mammalian cells, with specificity for ssDNA, and acts by promoting the degradation of this genetic material, decreasing DNA detection in the cytosol [11,12]. The presence of free DNA in the cytoplasm is detected by cyclic GMP-AMP synthase (cGAS), inducing the process of antiviral responses mediated by IFN-I [13]. However, the marked activation of this pathway can trigger autoimmune diseases. Therefore, the action of TREX-1 is important to control, in part, the development of autoimmune reactions [12]. Yang et al. (2007) demonstrated that cells with reduced TREX-1 activity failed to recognize and eliminate ssDNA, stimulating autoimmune dysfunction [14]. TREX-1 activation deficiency resulting from mutations in the gene is responsible for the abnormal accumulation of cytosolic DNA and for the stimulation of an intense and chronic proinflammatory response mediated by INF-I [15,16]. Alterations in the *TREX1* gene (which reduce enzyme activity) have been associated with Aircardi Goutieres Syndrome, retinal vasculopathy with cerebral leukodystrophy and lupus [17,18,19].

HIV converts its genetic material from ssRNA to dsDNA before integration into the host cell genome, so the virus’s DNA can also be targeted by TREX-1. This exonuclease is considered a factor of viral permissiveness because when it degrades the HIV-1 DNA, the IFN-I production pathway, responsible for efficient antiviral response, is attenuated, resulting in a productive infection; the absence of TREX-1 induces a potent IFN-I-mediated immune response [20]. In this regard, the action of TREX-1 seems to contribute to camouflaging the presence of HIV-1 to the recognition of DNA sensors, inhibiting the intracellular recognition of the virus and the consequent antiviral responses against the infection, resulting in a higher viral load [21,22]. The evaluation of certain polymorphisms in the *TREX1* gene showed an association with faster progression of HIV-1 infection [23].

Since the duration of ART use and variations in TREX-1 activity have been related to the development of autoantibodies, which can induce complications during HIV-1 infection, the present study performed a cross-sectional evaluation and longitudinal analysis to investigate the association between the presence of autoantibodies and the duration of ART use in people living with HIV-1. Furthermore, this was the first study that investigated the correlation between the TREX1 531C/T polymorphism and the presence of ANA in HIV-1 infection. The results of the study may provide a better understanding of factors that may influence the production of autoantibodies and the evolution of the infection.

## 2. Results

The epidemiological and treatment characteristics of the individuals involved in the study are described in Table 1. The variables male, single, family income of 1 to 3 minimum wages and high school education were the most frequent in the three groups evaluated; the average age of the group of individuals without ART was lower when compared to the other groups. In the group that used ART for 10 years, a greater variation in the types of therapeutic schemes was observed, which also showed a higher frequency of changing the therapeutic scheme.

In the evaluation of genotype frequencies for the *TREX1* 531C/T polymorphism and ANA positivity, no significant differences were observed between the groups of individuals with HIV-1 and the control group. However, plasma levels of IFN-α were higher in the control group (*p* < 0.0001; Table 2).

**Figure 1:**
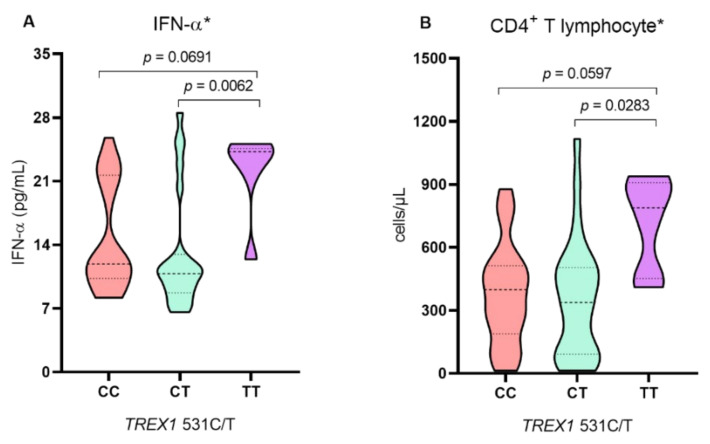

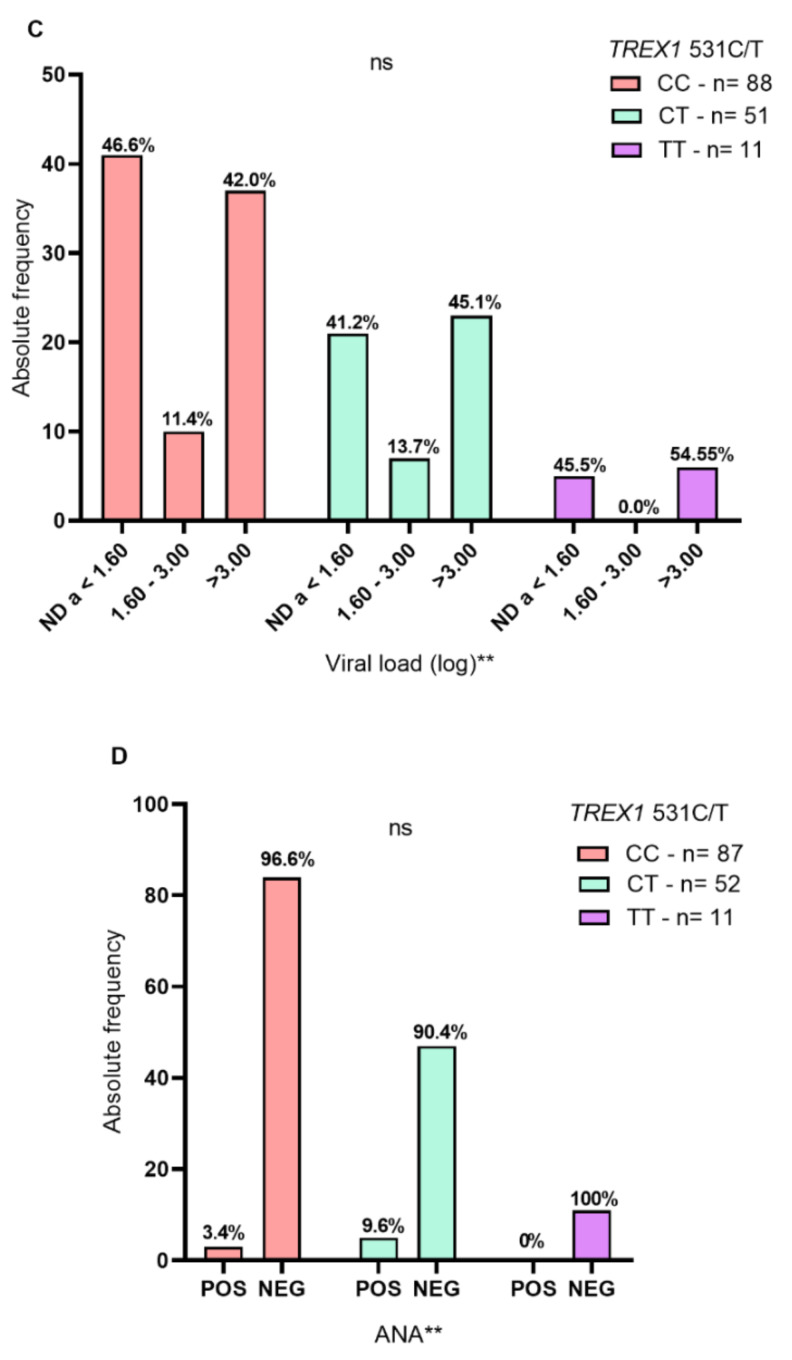
Comparison of levels of (**A**) IFN-α, (**B**) CD4^+^ T lymphocytes, (**C**) viral load and (**D**) ANA frequency according to genotypes for the *TREX1* 531C/T polymorphism. * Kruskal–Wallis test; ** G test, ns: not significant.

The levels of CD4^+^ T lymphocytes, IFN-α, viral load and positivity for ANA were evaluated in relation to the genotypes for the *TREX1* 531C/T polymorphism and demonstrated that the levels of CD4^+^ T lymphocytes and IFN-α were higher in individuals with the TT polymorphic genotype (*p* < 0.05; Figure 1A,B), but no significant difference was observed in viral load levels (log 10) (Figure 1C) or ANA frequency (Figure 1D) between the different genotypes for the *TREX1* 531C/T polymorphism.

**Figure 2:**
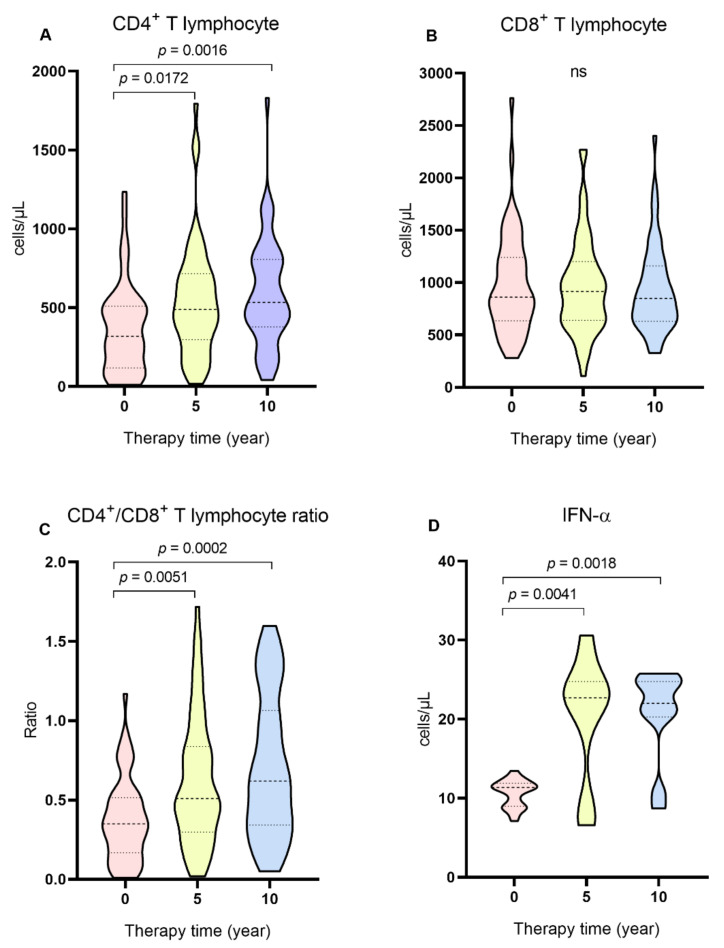
Comparison of the levels of (**A**) CD4^+^ T lymphocytes, (**B**) CD8^+^ T lymphocytes, (**C**) CD4^+^/CD8^+^ ratio and (**D**) IFN-α between groups naïve to ART, with 5 and 10 years of therapy. Kruskal–Wallis test, ns: not significant.

In the cross-sectional evaluation, the groups undergoing ART (5 and 10 years of treatment) had a significantly higher frequency of ANA (Table 3), higher levels of CD4^+^ T lymphocytes (Table 3 and Figure 2A), higher CD4^+^/CD8^+^ ratio (Table 3; Figure 2C) and IFN-α (Table 3; Figure 2D) compared to the ART naïve group. In contrast, groups with 5 and 10 years of ART had reduced levels of viral load (Table 3).

**Figure 3:**
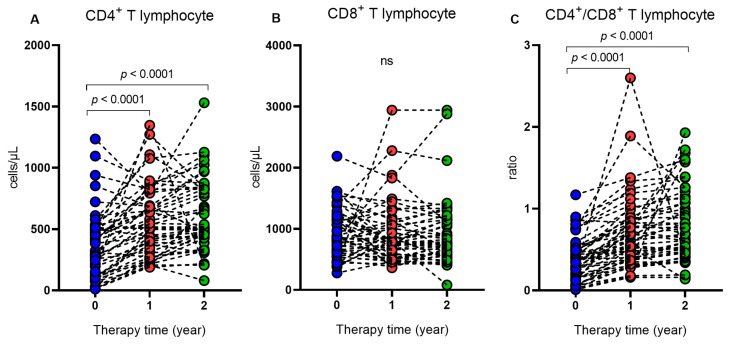
Comparison of the levels of (**A**) CD4^+^ T lymphocytes, (**B**) CD8^+^ lymphocytes and (**C**) CD4^+^/CD8^+^ ratio of individuals with HIV-1 in the period without ART and after one and two years of therapy, ns: not significant.

The longitudinal evaluation considered the moment when the individuals were naïve to ART and after these individuals underwent therapy for a period of one and two years. The results show that after one and two years of treatment, the individuals had a higher frequency of ANA, higher levels of CD4^+^ T lymphocytes (Table 4 and Figure 3A) and the CD4^+^/CD8^+^ ratio (Table 4; Figure 3C) and lower levels of viral load (Table 4) when compared to the period before starting therapy.

**Figure 4:**
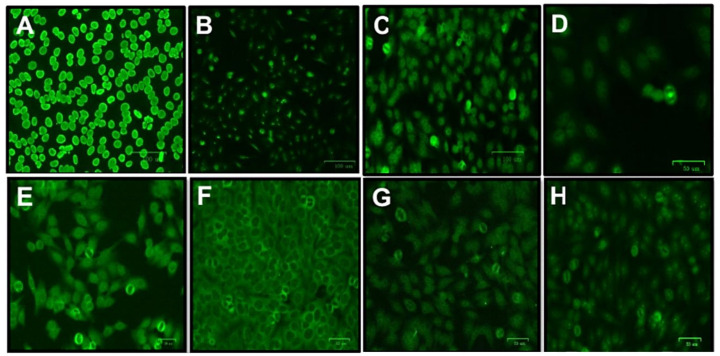
Immunofluorescence patterns of antinuclear antibodies in HEp-2 ANA+ cells from individuals with HIV-1 at different times of treatment. (**A**) homogeneous nuclear; (**B**) fine dotted nucleolar; (**C**) fine dotted nuclear; (**D**) Numa (mitotic apparatus); (**E**) cytoplasmic; (**F**) fine dotted cytoplasmic; (**G**) thick dotted nuclear; (**H**) nucleolar with isolated dots. Image resolution 100 µm (**A**–**C**), 50 µm (**D**–**H**).

The fluorescence patterns and therapeutic regimens of individuals with HIV-1 who were ANA positive are described in Table 5. In the cross-sectional evaluation, it was observed that individuals with 5 years of ART had a therapeutic regimen consisting mainly of tenofovir + faldaprevir + lamivudine (*n* = 3; 75%) and cytoplasmic, nuclear, and Numa fluorescence patterns; among individuals with 10 years of ART, 50% (*n* = 2) used tenofovir + lamivudine + efavirenz and had nuclear and nucleolar fluorescence patterns. In the longitudinal analysis, all ANA-positive individuals used the same therapeutic regimen at one and two years of ART, which consisted of tenofovir + lamivudine + efavirenz (*n* = 9; 100%). Individuals with 1 year of ART showed patterns of nuclear and nucleolar fluorescence; individuals with 2 years of ART showed different patterns (nuclear, nucleolar, cytoplasmic and Numa). The fluorescence patterns found in samples of individuals with positive ANA and the therapeutic schemes are shown in Figure 4.

## 3. Discussion

It is unquestionable that ART promotes improvements in the survival and life expectancy of individuals with HIV; however, the need for treatment for an indefinite period may result in the development of complications for some individuals living with the virus [24], including adverse effects, metabolic changes and autoimmune manifestations [3,4,6,7,25].

In the cross-sectional evaluation, the study showed that HIV-1 infection was not associated with the *TREX1* 531C/T polymorphism or the presence of autoantibodies, but individuals with HIV-1 had significantly reduced levels of IFN-α. Although variations in TREX-1 activity influence the course of the inflammatory response [12,14,16], the presence of the *TREX1* 531C/T polymorphism does not influence susceptibility or protection against HIV-1. Booiman et al. (2014) reported that the *TREX1* 531C/T polymorphism had no effect on the progression of HIV-1 infection [23], while the study by Pontillo et al. (2013) described that there was no difference in the frequency of the polymorphism between individuals infected with HIV-1 and a control group, but the authors did not assess the progression of the infection [26]. Although these studies did not observe an association between the *TREX1* 531C/T polymorphism and HIV-1 infection, they did not assess the polymorphism in relation to components of the inflammatory/antiviral response, such as IFN-α, nor with the presence of ANA, parameters included in the present study.

Genotype analysis for the *TREX1* 531C/T polymorphism among individuals with HIV-1 showed that carriers of the TT polymorphic genotype had higher levels of CD4^+^ T lymphocytes and IFN-α. Since the increased TREX-1 activity may impair IFN-α activation, the reduced exonuclease activity allows for more intense IFN-α production [12]. Furthermore, considering the importance of IFN-α in innate immunity as responsible for inducing an antiviral state and for contributing to the activation of the adaptive immune response [27], the results of the present study suggest that the TREX1 531C/T polymorphism may influence TREX-1 activity; the homozygous polymorphic genotype seems to contribute to better maintenance of the immune status, even if for a limited period of time.

In contrast, there was no difference in viral load levels or ANA frequency among individuals with different genotypes for the TREX1 531C/T polymorphism. Although TREX-1 promotes retroviral DNA degradation and influences the immune response and susceptibility to HIV infection [12,26], its exonuclease activity is not sufficient to control established infection. Thus, the *TREX1* 531C/T polymorphism is not associated with significant variations in HIV-1 viral load levels. The analysis of the *TREX1* 531C/T polymorphism in HTLV-1 infection showed that individuals with polymorphic genotypes had higher proviral loads, a result that suggested an influence of the polymorphism on TREX-1 activity, inducing lower immune control, which would promote maintenance of HTLV-1 infection [28]. Although HIV-1 and HTLV-1 are retroviruses, they have biological properties that differ from each other, mainly related to the viral replication capacity, reflected in higher levels of HIV-1 viral load and lower levels of HTLV-1 proviral load [29]. Thus, the influence of the *TREX1* 531C/T polymorphism may be different in these two types of retroviral infections.

The cross-sectional analysis of the duration of ART use showed that individuals who were on treatment for a period of 5 and 10 years had a higher frequency of ANA, higher levels of CD4^+^ T lymphocytes, a higher ratio of CD4^+^/CD8^+^ T lymphocytes, lower levels of viral load and higher levels of IFN-α than therapy-naïve individuals. In the longitudinal evaluation, individuals with 1 and 2 years of ART use showed variables with similar characteristics (higher frequency of ANA, higher levels of T CD4^+^ lymphocytes, higher ratio of T CD4^+^/CD8^+^ lymphocytes and lower levels of viral load) in relation to the period that they still did not undergo the treatment. The production of autoantibodies in HIV-1 infection and the use of ART is not yet well established, as some studies show divergent results. HIV-1-induced infection was associated with the production of autoantibodies since ART-naïve HIV patients had a higher prevalence of autoantibodies compared to patients with HIV undergoing treatment. The authors suggested that the immune dysregulation caused by the infection leads to the autoimmune manifestation, mediated by the action of CD33 + CD11b + HLA-DR+ cells and that the frequency of these cells decreased with the use of HAART [30]. Sedlacek et al. (2003) also described that the use of ART promoted a decrease in the levels of antiphospholipid antibodies, but some individuals still maintained the presence of these antibodies after five years of treatment [6]. Among infected individuals undergoing treatment, the presence of autoantibodies was associated with individuals with lower levels of CD4^+^ T lymphocytes [31]. In contrast, other studies have shown that a higher frequency of autoantibodies has been associated with the use of ART [7,32]. Iordache et al. (2014) described that ART promotes better immunological control of people infected by HIV-1 and the development of different autoimmune diseases [7]. Autoimmune manifestations of the thyroid can occur after the immune recovery resulting from the use of ART, which can induce the production of altered T lymphocytes, triggering autoimmunity through the deregulation of peripheral tolerance [32].

Differences in results between studies may be related to the stage of HIV-1 infection, as proposed by Zandman–Goddard and Shoenfeld (2002), in which autoimmune manifestations may be present, especially during the period of acute infection (when the immune system is still competent) and during ART-mediated immune restoration [5]. In the present study, the presence of ANA was not observed in HIV-1 individuals without ART. This result may be related to the impossibility of defining the time of infection of individuals who had been recently diagnosed; consequently, it is not possible to identify the stage of infection in these individuals.

The higher frequency of ANA observed in the period of recovery of immune competence induced by the use of ART corroborates previous findings that associated this immune recovery with the development of autoimmune diseases [7,31]. ANA identification was used to identify individuals with HIV-1 who may be at risk for developing autoimmune diseases. Although the presence of ANA has been observed in individuals with different durations of ART use, most of these individuals do not maintain autoantibody production, demonstrating that in these cases, the presence of ANA is not continuous. In addition, most individuals who had ANA were treated with tenofovir and lamuvudine, but these drugs were not associated with certain patterns of ANA. These results indicate that ANAs are associated with an improvement in the immune status, but in most cases, production occurs transiently and regardless of the treatment used. In this sense, carrying out a screening of individuals at the beginning and after some time of ART would be important to identify those who present a real risk of developing an autoimmune disease.

## 4. Materials and Methods

### 4.1. Type of Study, Population and Sample Collection

The study included two types of assessments: one cross-sectional and one longitudinal. The cross-sectional study consisted of analyzing 150 people living with HIV-1, divided into three groups: naïve to ART (*n* = 50), with 5 years of ART use (*n* = 50) and with 10 years of ART use (*n* = 50) (Table 1). The longitudinal evaluation consisted of forming a cohort of 50 ART-naïve individuals with HIV-1 who were followed up and evaluated after one and two years of treatment use. The individuals evaluated in the study came from the outpatient clinic of CTA/SAE—Casa Dia, located in the city of Belém, Pará, Brazil. The inclusion and exclusion criteria are described in Table 6.

A comparison group was formed by samples of 100 sex workers who were seronegative for HBV, HCV, HIV, HTLV, Chagas disease, syphilis, and age- and sex-matched with individuals with HIV-1 was used to compare genotype frequencies for TREX1 531C/polymorphism T. This group was referred to as the control group.

Blood samples were collected in two tubes, one with EDTA anticoagulant (5 mL) and the other without anticoagulant with separator gel (5 mL). The samples were transported to the Laboratory of Virology at the Federal University of Pará, where they were processed to separate aliquots of whole blood, serum and plasma. Whole blood samples were used for LTCD4^+^/LTCD8^+^ quantification and DNA extraction; plasma samples were used to measure HIV-1 plasma viral load and IFN-α dosage, and serum samples were used for ANA research.

### 4.2. ANA Research in HEp-2 Cells

The samples were tested using the VIRGO^®^ ANA/HEp-2 IgG indirect fluorescent antibody (IFA) kit (Hemagen Diagnostics, Columbia, MD, USA) following the manufacturer’s specifications. The slides were read using an Eclipse E-200 immunofluorescence microscope (Nikon, Minato, Tokyo, Japan) using 10X and 40X ocular lenses. To record and store the images obtained from the fluorescence patterns, a fluorescence microscope ZOE Fluorescent Cell Imager (Bio-Rad, Hercules, CA, USA) was used.

### 4.3. Plasma Dosage of IFN-α

Quantification of IFN-α levels was performed by enzyme-linked immunosorbent assay (ELISA) using the IFN alpha Human ELISA kit (Thermo Fisher, Waltham, MA, USA), which uses specific monoclonal antibodies to detect the cytokine. The test was performed following the manufacturer’s recommendations.

### 4.4. DNA Extraction

DNA was extracted using the total DNA extraction method from peripheral blood cells following the steps of cell lysis, protein precipitation, DNA precipitation and DNA hydration using the Puregene™ kit (Gentra Systems, Minneapolis, MS, USA). All process steps followed the manufacturer’s recommendations. The DNA obtained was quantified by spectrophotometric reading in BioDrop™ equipment (Bio-Rad, Hercules, CA, USA) following the protocol recommended by the manufacturer.

### 4.5. TREX1 531C/T Genotyping (rs11797)

Genotyping was performed by real-time PCR using the StepOnePLUS™ Real-Time PCR System (Thermo Fisher, Carlsbad, CA, USA). The reaction was performed using the TaqMan™ TREX1 rs11797 assay (C__11537906_20), commercially obtained, containing specific primers and probes for amplification of the target sequence (Thermo Fisher, Carlsbad, CA, USA). The reaction consisted of 1x MasterMix, H_2_O, 20x assay C_11537906_20 and 50 ng of DNA. The cycling program was 10 min at 95 °C and 40 cycles of 15 s at 95 °C and 1 min at 60 °C.

### 4.6. Quantification of TCD4^+^/TCD8^+^ Lymphocytes and HIV-1 Plasma Viral Load

The TCD4^+^ and TCD8^+^ lymphocyte counts were performed at the Virology Laboratory, as established by the National Network of CD4^+^/CD8^+^ T lymphocytes of the Ministry of Health. Flow cytometry methodology was used, which evaluated a whole blood sample with EDTA, using BD FACScount equipment and BD multitest reagents (CD45, CD3, CD4 and CD8) (BD, Franklin Lakes, NJ, USA), following the protocol recommended by the manufacturer.

Plasma viral load was determined at the Virology Laboratory, following the standard methodology of the National Viral Load Network of the Ministry of Health, based on real-time PCR technology, using the Sample Purific CV HIV-1 Extraction Kit and the HIV-1 Viral Load Amplification and the Abbott 2000 mrt thermal cycler (ABBOTT, Chicago, IL, USA) and all steps were followed by the manufacturer’s recommendations. Viral load measurements were used in log10.

### 4.7. Statistical Analysis

The information obtained was entered into a database using Microsoft Office Excel 2013 software. All tests were performed using the GraphPad Prism 8.0 program, with significant associations being those with a *p* value < 0.05.

Allelic and genotypic frequencies of polymorphisms were determined by direct counting, and differences between groups were evaluated using the G test. The Hardy–Weinberg equilibrium test was performed to assess the expected frequency of TREX1 531C/T genotypes in each group. The normal distribution of quantitative data was performed using the Shapiro–Wilk test. In the cross-sectional evaluation, the nonparametric Mann–Whitney and Kruskal–Wallis tests were used, and in the longitudinal evaluation, the Friedman test was performed.

## 5. Conclusions

The study showed that the *TREX1* 531C/T polymorphism does not influence ANA production, but the polymorphism was associated with better maintenance of the immune status of individuals with HIV-1, suggesting that the polymorphism may induce lower TREX-1 activity. ANA production was related to immune restoration in individuals with different durations of ART use, indicating the need for autoantibody screening to identify individuals with HIV-1 who are at risk of developing an autoimmune disease.

## Figures and Tables

**Table 1:** Characteristics of the different groups of individuals with HIV-1 evaluated.

Characteristics	ART-Naïve *n* = 100 *n* (%)	5 Years ART *n* = 50 *n* (%)	10 Years ART *n* = 50 *n* (%)
**Sex**			
Female	27 (27.0)	22 (44.0)	19 (38.0)
Male	73 (73.0)	28 (56.0)	31 (62.0)
**Age**; mean (SD)	29.8 (9.7)	40.5 (10.2)	44.6 (12.3)
**Marital status**			
Married	34 (34)	15 (30)	14 (28)
Single	66 (66)	28 (56)	30 (60)
Divorced	0 (0)	4 (8)	1 (2)
Widower	0 (0)	3 (6)	5 (10)
**Family income (minimum wage)**			
<1	16 (16)	8 (16)	7 (14)
1 a 3	65 (65)	32 (64)	37 (74)
≥4	14 (14)	9 (18)	5 (10)
No information	5 (5)	1 (2)	1 (2)
**Education**			
Elementary School	16 (16)	14 (28)	14 (28)
High school	65 (65)	27 (54)	28 (56)
University education	19 (19)	9 (18)	8 (16)
**Therapeutic scheme**			
Etravirine + Ritonavir	NA	0 (0)	1 (2)
Maraviroc + ritonavir + darunavir + dolutegravir	NA	0 (0)	1 (2)
Tenofovir + atazanavir + lamivudine + ritonavir	NA	4 (8)	2 (4)
Tenofovir + Atazanavir + ritonavir	NA	2 (4)	0 (0)
Tenofovir + lamivudine + efavirenz	NA	24 (56)	15 (30)
Tenofovir + lamivudine + dolutegravir	NA	0 (0)	0 (0)
Tenofovir + lamivudine + ritonavir + darunavir	NA	1 (2)	0 (0)
Tenofovir + lamivudine + lopinavir + ritonavir	NA	0 (0)	6 (12)
Tenofovir + abacavir + didanosine + lamivudine + zidovudine + efavirenz + nevirapine + etravirine	NA	0 (0)	2 (4)
Tenofovir + Lamivudine + atazanavir	NA	0 (0)	3 (6)
Tenofovir+ Raltegravir + Lamivudine	NA	3 (6)	0 (0)
Tenofovir + lamivudine + efavirenz + ritonavir + darunavir	NA	0 (0)	1 (2)
Tenofovir + lamivudine + Efavirenz + ritonavir + Darunavir	NA	0 (0)	1 (2)
Zidovudine + Lamivudine + lopnavir + ritonavir	NA	3 (6)	1 (2)
Zidovudine + lamivudine + efavirenz	NA	12 (24)	10 (20)
Zidovudine + didanosine + Efavirenz	NA	0 (0)	1 (2)
Zidovudine + lamivudine + atazanavir	NA	0 (0)	1 (2)
Zidovudine + lamivudine + atazanavir + ritonavir	NA	0 (0)	3 (6)
Zidovudine + lamivudine + nevirapine	NA	0 (0)	2 (4)
Zidovudine + lamivudine + daranavir + ritonavir + dolutegravir		1 (2)	0 (0)
**Change of therapy**			
No	NA	28 (56)	11 (22)
Yes	NA	22 (44)	39 (78)

ART: antiretroviral therapy; NA: not applicable; SD: standard deviation.

**Table 2:** Evaluation of genotype frequencies for *TREX1* 531C/T, ANA positivity and IFN-α levels between individuals with HIV-1 and the control group.

Variables	HIV-1 *n* = 150 *n* (%)	Control *n* = 100 *n* (%)	*p*
***TREX1*** **531C/T** **Genotypes and alleles**			
CC	88 (58.7)	60 (60)	0.8367 *
CT	51 (34)	31 (31)	
TT	11 (7.3)	9 (9)	
C	0.76	0.75	0.9492 *
T	0.24	0.25	
**ANA**			
Positive	8 (5.3)	1 (1)	0.1231 *
Negative	142 (94.7)	99 (99)	
**IFN-α**, median (IQR)	11.3 (12.2)	22.7 (6.88)	<0.0001 **

ANA: antinuclear antibody; *n*: number of individuals; IQR: interquartile range. * G test; ** Mann–Whitney Test.

**Table 3:** Evaluation of ANA frequencies and levels of T lymphocytes, viral load and IFN-α among individuals with HIV-1 naïve to ART, with 5 and 10 years of ART.

Variables	ART-Naïve *n* = 50 *n* (%)	5 Years ART *n* = 50 *n* (%)	10 Years ART *n* = 50 *n* (%)	*p*
**ANA**, *n* (%)				
Positive	0 (0)	4 (8)	4 (8)	0.0452 *
Negative	50 (100)	46 (92)	46 (92)	
**CD4^+^**, median (IQR)	319 (391.2)	489.5 (418.7)	534.5 (428.3)	0.0012 **
**CD8**^+^, median (IQR)	862 (607.2)	915.5 (560.2)	848 (529.5)	0.8685 **
**CD4^+^/CD8^+^**, ratio (IQR)	0.35 (0.3)	0.51 (0.5)	0.62 (0.7)	0.0001 **
**Viral load**, log10				
ND a < 1.60	1 (2)	31 (62)	36 (72)	<0.0001 *
1.60–3.00	1 (2)	9 (18)	5 (10)	
>3.00	48 (96)	10 (20)	9 (18)	
**IFN-α**,median (IQR)	11.36 (2.91)	22.7 (10.36)	22.01 (4.47)	0.0002 *

ART: antiretroviral therapy; ANA: antinuclear antibody; *n*: number of individuals; IQR: interquartile range; ND: not detected. * G test; ** Kruskal–Wallis test.

**Table 4:** Assessment of ANA frequencies, T lymphocyte levels and viral load in individuals with HIV-1 in the period without ART and after one and two years of therapy.

Variables	ART-Naïve *n* = 50 *n* (%)	1 Year ART *n* = 50 *n* (%)	2 Years ART *n* = 50 *n* (%)	*p*
**ANA**, *n* (%)				
Positive	0 (0)	3 (6)	6 (12)	0.0174 *
Negative	50 (100)	47 (94)	44 (88)	
**CD4^+^**, median (IQR)	314 (394)	517 (410)	509 (393.5)	<0.0001 **
**CD8^+^**, median (IQR)	852.5 (585)	778 (579.7)	782 (540.5)	0.3966 **
**CD4^+^/CD8^+^**, ratio	0.35 (0.4)	0.61 (0.5)	0.69 (0.6)	<0.0001 **
**Viral load**, log 10				
ND a < 1.60	1 (2)	42 (84)	44 (88)	<0.0001 *
1.60–3.00	1 (2)	5 (10)	5 (10)	
>3.00	48 (96)	3 (6)	1 (2)	

ART: antiretroviral therapy; ANA: antinuclear antibody; *n*: number of individuals; IQR: interquartile range; ND: not detected. * G test; ** Friedman test.

**Table 5:** Description of fluorescence patterns and therapeutic schemes of individuals with HIV-1 positivity for ANA.

Patient (ID)	Fluorescence Patterns	Therapeutic Scheme
**Cross-sectional evaluation**		
**5 years ART**		
27984	Fine dotted cytoplasmic	Tenofovir + raltegravir + lamivudine
27987	Nucleolar	Tenofovir + raltegravir + lamivudine
28273	Numa (mitotic apparatus with positive poles)	Tenofovir + raltegravir + lamivudine
28617	Thick dotted nuclear	Zidovudine + lamivudine + darunavir + ritonavir + dolutegravir
**10 years ART**		
26714	Fine dotted nuclear	Tenofovir + lamivudine + efavirenz
26733	Homogeneous nuclear	Tenofovir + lamivudine + atazanavir
26862	Nucleolar	Tenofovir + Abacavir + didanosine + lamivudine + zidovudine + efavirenz + nevirapine + etravirine
29947	Thick dotted nuclear	Tenofovir + lamivudine + efavirenz
**Longitudinal evaluation**		
**1 year ART**		
26468	Fine dotted nuclear	Tenofovir + lamivudine + efavirenz
26541	Fine dotted nuclear	Tenofovir + lamivudine + efavirenz
26712	Nucleolar with isolated dots	Tenofovir + ritonavir + atasanavir
**2 years ART**		
26468	Thick dotted nuclear	Tenofovir + lamivudine + efavirenz
26471	Fine dotted nuclear	Tenofovir + lamivudine + efavirenz
26480	Dotted nucleolar	Tenofovir + lamivudine + efavirenz
26536	Dotted nucleolar	Tenofovir + lamivudine + efavirenz
26537	Fine-speckled nuclear and Numa (mitotic apparatus)	Tenofovir + lamivudine + efavirenz
26703	Cytoplasmic	Tenofovir + lamivudine + efavirenz

ART: antiretroviral therapy.

**Table 6:** Inclusion and exclusion criteria used to select individuals with HIV-1 from the evaluated groups.

Groups	Inclusion Criteria	Exclusion Criteria
ART-naïve	Age ≥ 18 years old, both sexes, never having used antiretrovirals.	Diagnosis of autoimmune disease, coinfection with Hepatitis B Virus, Hepatitis C Virus and Hepatitis D Virus and HTLV-1, use of corticosteroids and clinical diagnosis of AIDS.
5 years ART	Age ≥ 18 years, both genders, having been on treatment (ARV) for a period of five years without interruptions	
5 years ART	Age ≥ 18 years, both genders, having been on treatment (ARV) for a period of 10 years without interruptions.	

ARV: antiretrovirals.

## Data Availability

The data analyzed in this study are included in the paper.
